# Bibliographical Notices

**Published:** 1838-10-01

**Authors:** 


### Periscope;

OR,

### CIRCUMSPECTIVE REVIEW.

"Ore trahit quodcunque potest, atque addit acervo."

### Notices of some New Works.

The Influence of Religion on Health and the Physical Welfare of Mankind. By *Amariah Brigham*, M.D. Boston.

Man is, by Nature, a religious animal, but since the first creation down to the present moment, superstition has been to true religion as ten thousand to one. The miseries engendered by the *former*, in all ages and countries, have far more than counterbalanced the blessings that naturally flow from the *latter*. Even under the divine dispensation of Christianity, how much hatred, malice and all uncharitableness is daily put in action by the clashing of doctrines and creeds amongst the different sects of the same religion! It is a most melancholy and humiliating task to even glance at the sufferings to which frail humanity has been subjected by the credulity, prejudice, bigotry, and superstition of man himself! The ancient Egyptians did not shed the blood of animals on their altars, yet they sacrificed men to their gods—and every year a virgin to the Nile. Aristomenes, called the *Just*, slew three hundred noble Lacedæmonians at the altar of Jupiter at Ithome! The Lacedæmonians could not complain, as they offered victims to Mars. The Romans were not behind the Greeks in this respect. Many of the captives were sacrificed to Jupiter Capitolinus—and Caius Marius sacrificed his only daughter, Calphurnia, to insure a victory over the Cimbri. Augustus Cæsar immolated three hundred chosen men, on the Ides of March, at an altar dedicated to the manes of his uncle Julius! The Gauls and Germans were as bloody as any of the ancients. The horrors of these sacrifices drew from Plutarch a remarkable passage, the abbreviated purport of which may thus be stated. "Would it not have been better for these nations to have had no traditions or conceptions of gods at all, than to have worshipped deities who esteemed human victims the most acceptable offerings, and who delighted in the blood of man?" If we range over the historic page to the far West, we find proofs of human sacrifices from Terra del Fuego to the Falls of Niagara; and when, weary and disgusted with these degrading spectacles, we turn to the glowing Orient, and survey the vast plains and snow-clad mountains of China and Hindostan, we see nothing consolatory—especially in the latter. Even to this hour, the wheels of Jaggernaut crush the bodies and bones of hundreds of human beings—the burning pile receives the shuddering widow—and the aged and infirm are carried down to the shores of the Ganges, where they are deposited at low-water mark, that the waves of the holy river may flow over them, and bear the immortal spark to the ocean, whence it may fly back and be absorbed into the essence or deity from which it was first emitted!

Time and the growth of reason have obliterated these immolations from most nations, and are diminishing them in all; yet still, even in the most civilized Christian states, there are certain rites, ceremonies, observances—but especially feelings and opinions, which may well occupy the attention of the medical philosopher, as bearing on health and disease. We shall glance briefly at some of these which are considered in detail by Dr. Brigham.

1. *Circumcision.*—There is no doubt but that this ceremony was, at first, not a religious one. At all events it was practised by the ancient Egyptians, long before the introduction of Judaism or Christianity. That the practice is salutary in hot climates we believe; but we are far from thinking it beneficial in cold countries.

2. *Castration.*—Was a religious ceremony amongst the priests of Cybele and of Atys. Moses, however, prohibited it, and the Mahomedans continue it for other than religious reasons. It is curious that it seems to be sanctioned in the 19th Chapter of St. Matthew—and the great Origen and some of his followers emasculated themselves! The influence of this mutilation on the morale and physique of man is well known.

We shall pass over the *flagellation* of the ancient Christians—the cuttings and maimings of the Persians, Hindoos, &c., and come to the 3d Chapter, on

*Monachism and its Austerities.*—Waddington remarks that the monastic spirit was congenial to the scenery and climate of the East, and to the peculiar character of the inhabitants. Vast solitudes of unbroken and unbounded expanse—rocks with strange and grotesque outlines—while a dry air and unclouded sky offered facilities, if not temptations, to a wild, contemplative, and unsocial life. It was in the early part of the fourth century, however, that Christian Monacism became established—and by the middle of the sixth century monkery had so increased that, according to Mosheim, "whole armies of them might have been enrolled, without any sensible diminution of their numbers." A Spanish writer states that he counted in Spain 47,000 abbeys, 40,000 priories, and 14,000 convents!

"I hardly need add that this kind of life is injurious to the health, both of body and mind. Medical writers enumerate many diseases to which these religious recluses, of both sexes, were peculiarly liable. Besides, a life thus passed, contrary to the wishes of nature, has the effect to render the character austere, despotic, and often melancholy; and at the same time, disposes to exaltation and excitement of the brain and nervous system, and gives rise to innumerable nervous complaints."

In by-gone days, when the priests led hermits' lives or kept cloistered in their monasteries, the austerity in question did, no doubt, obtain; but now the men of God mix with society, partake of all the good things of this life—except matrimony—and enter not a little into the feelings, political and social, of mankind; so that, in fact, they are little distinguishable from other people, except by their canonicals in the chapel.

Of *fasting* little need be said. Like most other religious injunctions, it was designed for health. The fast of the Ramadam, for instance, is observed during the hottest season of the year, and its effects were, doubtless, beneficial. The spare diet of the Hindoo, and the prohibition of animal food, may be referred to the same origin. The Lent of the Catholics tends to keep them in health, and it would be curious to know whether that class of religionists is longer lived than Protestants or other sects.

The fourth chapter investigates the influence on health of certain religious rites and ceremonies, as the Lord's supper—baptism—places of worship—night-meetings, &c. for which we must refer to the work itself, as containing a great deal of curious and interesting information.

In the 7th Chapter our author discusses the injuries to the brain and nervous system from the excitement and depression resulting from religious meetings and religious meditations. This is an exceedingly valuable chapter, and we wish it could be perused by all clergymen, especially the evangelical ones—and all those who think, or seem to think, that our Creator sent us into this world for no other purpose than to weep and wail, and mortify the flesh for the salvation of our souls. Yet such would not appear to be the design of the Omnipotent Being who was the author of our existence. He would not have endowed man with organs and capacities for the enjoyment of all Nature's gifts, had he not intended that these gifts should be enjoyed.

The work concludes with a short chapter on the utility of the Sabbath as a day of rest, religious duties, and enjoyment—not of gloomy fanaticism, austerity, and privation.

"But if the divine command given in the Old Testament, on this subject, does not oblige us to keep this day, then we have none whatever. Christ has not given us any. Still I think he must have approved of it as a day of rest, for he found such an institution to exist, and he said nothing against it in this respect, though he condemned the strict Jewish observance of it. When charged with not observing it himself, he replied that 'it was lawful to do well on the sabbath days.' But it should be borne in mind, that he constantly taught that it was necessary to do well on all days. It is pitiful to see the efforts of some men to belittle this subject, and misunderstand and misrepresent the Christian duty in relation to the sabbath, and endeavour to make mankind believe that it is an offence against God to walk, ride, visit friends, or even to abstain from hearing sermons on that day. They seem not to be able to comprehend the difference between the teachings of Moses and Christ. While the former established rites and ceremonies, the latter sought only to create in man the sincere love of his Creator and of his fellow men. The high aim of Christ was to make men *do well* on all days, not on one particular day—to establish the love of God and man in the heart of man, not to establish a particular form of worship. Such was the great purpose of our Saviour—and thus it happened that the early Christians abandoned the Jewish sabbath, and established another devoted to rest, enjoyment, and deeds of charity and love." 316.

With this sensible extract, which we would recommend to the perusal of Mr. Plumptre and Sir Andrew Agnew, we take leave of our author, with every sentiment of respect for his talents and erudition.

---

Buxton and its Waters—An Analytical Account of their Medicinal Properties and General Effects. By *W. H. Robertson*, M.D. Duodecimo, pp. 147. London, 1838.

England is not blessed with those numerous subterranean fires which boil the water and warm the air for our Continental neighbours. Our latent volcanos—few and far between—are also upon an insignificant scale, compared with those in France, Spain, Italy and Germany. No wonder that our climate is cold, wet, foggy, and variable—and to crown our misfortunes we are doomed to drag out a protracted, though vigorous existence, beneath our gloomy skies, of from ten to fifteen years longer than the blessed inhabitants of the most favoured climate in the world. Nature is a step-mother. She orders her inexorable executioner to mow down with his scythe a thirtieth part of mankind annually from the lovely vales of Italy, where existence is a kind of Paradise, but only sweeps away a fortieth or fiftieth of those ill-fated islanders—"toto orbe divisos" —doomed to breathe a misty atmosphere, and bide the pelting of the pitiless storm.

But to our work. Bath, Bristol, Buxton and Matlock, are, we believe, the only thermal springs of any note, in England. The Romans, who considered the warm bath as constituting one third of all that was worth living for—Balnea, Vina, Venus—appear to have smelled out Buxton, since many of their utensils and roads have been discovered there. It is situated on a high cold ridge, a thousand or more feet above the level of the sea, and sends forth a thermal spring in abundance, at a temperature of about eighty degrees of Fahrenheit. The analysis given by Sir Charles Scudamore, and conducted by Mr. Garden, is the most recent, and probably the most correct. There are about 15 grains of solid matter in the gallon—two thirds of which consist of carbonate of lime. There are also about six cubic inches of gazeous matters in the same quantity. The other ingredients are very small portions of muriate of magnesia, soda, and sulphate of lime. Like the baths of Pfeffers and Wildbad, there is a constant stream of water, and consequently an equality of temperature preserved.

"How little is to be learned as to the effects of these waters on disease, from a knowledge of their chemical composition and temperature, is fully shown by the following results, which have been gathered from the reports of the Buxton Bath Charity for the last eighteen years—from 1820 to 1837, both inclusive. Deducting the cases which have remained on the books at the time of making up every yearly report, from the total of the cases admitted during the year, and adding these cases to the total of the cases admitted and dismissed before the making up of the following year's report, it appears that 21,468 patients have been admitted on the books of this charity within the above-mentioned period. Of these, 6,562 have had medical advice, medicines, and the use of the baths, gratuitously. The remaining 14,906 cases have had the gratuitous use of the baths and medical advice, and been supplied with all necessary medicines, and received pecuniary relief to the amount of five shillings per week for the space of three weeks. Of these latter cases only, has an account been kept, as to the degree of benefit derived from the use of the waters. It appears that of the 14,906 patients, 12,608 have been dismissed 'cured or much relieved,' the remainder having been either little relieved, or no better at the time of dismissal. When it is remembered, that nearly the whole of this vast number of cases have been chronic cases, and that hardly any of them have been longer than three weeks in Buxton, the amount of relief afforded would be sufficient proof, if proof were wanting, that these waters are of powerful efficacy, and that the amount and character of their effects must be judged of by the results of cases and not by a mere reference to their chemical composition." 64.

After an exposition of the general physiological effects of bathing in non-medicinal waters, our author observes that the effects of the Buxton waters are different from those of common waters. If properly managed, they act as a sedative in cases of excitement—as a febrifuge in cases of fever. Their most remarkable effect is that of a stimulus. A man in health cannot bathe often in them with impunity. A person therefore must not be in a state of plethora, local or general, when he uses these waters—in short, he must be below par in health.

"In nearly every case, the effect produced by these waters, when used internally, unless in those whose systems from long use have become habituated to them, and unless the individual's powers are below the healthy standard, is *excitement*, an approach to feverishness; with the thirst, restlessness, &c., consequent thereupon. In most cases this yields to a dose of common aperient medicine, and it is seldom that any further inconvenience is occasioned by them, than may be relieved by having recourse occasionally to these simple means. Far different is it when these waters are used externally, in the form of bath, and as often as three or four times a week. The excitement thus produced is greater than exercise, or attention to diet, or regular hours, or repeated doses of opening medicines can subdue; and it is found necessary to desist from bathing, and in most cases to lower the system considerably, before the feverish state thus produced can be moderated, and health restored. These effects are all aggravated materially, if there had been a state of excitement present before the baths were used, or if the digestive organs had been deranged, or if any of the internal organs had been excited, or prone to excitement. Every year many cases of very serious illness occur, from a very few baths having been taken under such circumstances as these. How it happens, that a man who has used the shower bath, or the cold bath, or the tepid or hot bath, or who has been accustomed to bathe in the sea, or to use hot or tepid sea-water baths, and without injury, or perhaps with benefit, is thus excited and thrown out of health by bathing a few times in these waters, is a question which strikes at the root of the *modus operandi* of the Buxton waters; and this must be admitted to be quite inexplicable." 75.

Their temperature being 82° they, of course, communicate a chilly feeling on first immersion; but is followed, in a few minutes, by a glow of excitement, and the skin is speedily cleared of scurf and impurities, in consequence of the alkaline ingredients. The rheumatic patient usually loses his pains for a time—the debilitated invalid his sense of weakness—and the cripple his lameness. Their effects on the animal spirits are exhilarating.

"In from a few seconds to ten or twelve minutes, the individual leaves the water, with a delicious sense of warmth through his whole system, with a full consciousness of the unbounded enjoyment that attends the active discharge of the vital functions. Supposing him, as we have done, to be in a state of health, he finds, for the next twenty-four hours, that all his energies are augmented, that his appetite is sensibly increased, his digestion unusually active, his mind singularly free from despondence or irritability. This may be said to be the primary effect of the Buxton baths, they are immediately stimulating, not only to a much greater degree than ordinary water of the same temperature, but to a greater degree than any mineral water I have heard of." 77.

We fear Dr. Granville will feel a little jealous lest his magic bath of Wild-bad should be eclipsed by the more homely springs of Buxton.

If a man in health were to persevere with the Buxton waters, the exhilaration and excitement above described would change into a sluggish condition of the vital functions—torpor—and feverishness. Nothing but aperients and antiphlogistics would relieve these symptoms. But even where the system is in a fit state for the Buxton waters, and the disorders such as they generally cure, debilitating effects supervene upon the use of the waters after a certain time, and therefore they require watching. The duration of a course of the Buxton waters is exceedingly various. The average duration of the immersion is from four to twelve minutes—the average frequency of bathing every second or third day.

After what has been said of the properties of these waters, it will be evident to every medical reader that they are only proper in chronic affections, particularly rheumatism and gout, and where nothing like active inflammatory action is going on. Yet it is wonderful how many people are sent to these and other Spas with diseases the most opposite, and labouring under affections that must be injured not benefitted by the medicinal springs. With the following quotation we shall close our notice of this little work, which will prove very useful to the general as well as the professional reader.

"After what has been said of the effects of these waters, and of the states of system in which their use cannot but do harm, it will not fail to excite surprise that people should visit Buxton—many of them, I am sorry to say, by the recommendation of their medical attendants—for complaints the most dissimilar in nature, stage, and degree. Numbers of the unhappy victims of pulmonary consumption, numbers in an advanced stage of hepatic disease, are found every year among those who have come to make trial of these waters. Not long ago, I saw a case of affection of the head, following a blow on the occiput, and attended and probably kept up by irregular action of the heart, which required depletory measures to be had recourse to, that had been sent to try the baths of Buxton by a medical man who does not reside at a greater distance than thirty miles from this place. Cases of acute rheumatic inflammation of the joints are continually presented to us, which require an active course of local bleeding, blistering, &c., preparatory to a trial of the waters ; which treatment involves a considerable loss of time, sometimes puts the individual to serious pecuniary inconvenience, sometimes renders it impossible that he should give the baths a trial, by causing his scanty funds to be expended before he is in a fit state to use the baths, and which treatment might have been undergone at his home, with equal advantage.

So extensively do these evils operate, that in a very large proportion of the cases we meet with, the nature of which happens to be such as these waters are calculated to relieve or remove, a course of preparatory treatment before quitting home would have been of much use : and in very few cases has anything of the kind been done to an adequate degree—it might almost be said to any degree." 112.

---

PRACTICAL OBSERVATIONS ON HYSTERIA, ESPECIALLY RELATING TO ITS ORGANIC CHARACTER. By *John Prichard*, M.R.C.S., and Surgeon to the Leamington Hospital. pp. 36. Churchill, June 1838.

THE Proteus Hysteria has puzzled old and young in the profession, from the time of Hippocrates to that of Mr. Prichard. The experienced practitioner perhaps detects the disguised shapes which the malady assumes, while no small number of the younger branches mistake these shapes and forms for diseases of a different, often dangerous character. Sydenham attributed hysteria to "an irregular flow of animal spirits," which is saying nothing at all—but a great majority, we apprehend, of the faculty have taken the same view of its nature, or rather cause, as our author, namely—"uterine disturbance."

"This disturbance may be slight, and with difficulty detected, especially if we rely on the information which a cursory examination elicits from our patient. If the menstrual period returns with any thing like regularity, we are assured that all is right in that respect; but if we push our enquiries to the quantity of the discharge, its character, (whether pale or dark, fœtid or not, coagulated or otherwise,) the pain which accompanies it, especially on its accession, and the state of the uterus in the intervals, (whether affected by leucorrhœa, or by an occasional sanguineous discharge,) we shall probably arrive at evidence of functional disturbance, if not of structural mischief." 4.

If we admit that the slightest deviation from healthy uterine function furnishes adequate cause for hysteria, we shall seldom be at a loss for the existence of such cause. We shall, indeed, find this "uterine disturbance" in thousands of females who present no symptom of hysteria at all. Our author's theory will be gathered from the following passage.

"That the nervous system is the agent by which this sympathy is communicated, is now generally acknowledged ; and, in the production of hysteria, I am induced to regard its agency as effected in one of two ways—the operation of one or the other being determined by the state of the source of irritation, and producing a class of symptoms 'sui generis' in either case. I look upon the hysteric paroxysm, hysteric catalepsy, aphonia, dysphagia, &c., as originating in an excited state of an organ, the powers of which, intimately connected with the mind, are developed, and not exercised; and as communicated to the cerebrum, producing its immediate effect there, or thence transmitted to other parts of the nervous system, and especially to the par vagum and its important connexions. These affections occur for the most part to young females; but there is another period of life when the connexion between the cerebral and the uterine system gives rise to a train of vague and anomalous symptoms, no less alarming to the patient, than easy of relief by properly directed treatment; the periodical discharge, having perhaps duly returned for a space of thirty years, (excepting on occasions when the whole vascular energy of the uterus has, if I may so speak, been otherwise engaged,) abruptly ceases; the constitution, long accustomed to its influence, does not readily accommodate itself to the change, and the cerebral system soon partakes in the consequent disturbance. These affections then, whether the first, or the last named, I would place under the head of *Cerebral Hysteria*, in contra-distinction to those painful local affections, constituting another form of the disease, which I would call *Neuralgic Hysteria*; in these last cases the irritation seems to be communicated directly by that sympathy which one part is known to have with another, and, if I mistake not, will generally be found to arise from either diseased action, or disturbed function in the uterus. I am the more inclined to assume this distinction of the communicating media, from a consideration of the fact, that all the symptoms of the hysteric paroxysm may occasionally be witnessed in the male subject under the influence of cerebral excitement. I am willing however to admit, that these different states may, and do readily, lapse, the one into the other; or in other words, that the excited uterus is prone to assume diseased action, and that generally of an inflammatory character.

Thus then I would divide hysteria into two kinds, each arising from uterine irritation; but this irritation essentially differing in character in either kind, and the effect produced illustrating the character of the cause producing it.

With what I have ventured to name cerebral hysteria this enquiry has little to do; inasmuch as, originating in excitement merely, its effects generally end there; it is the Neuralgic form of the disease which more particularly bears upon the question under consideration. Before I proceed further, it will be well to refer to a modification of hysteria, by no means uncommon, and which does not seem to come precisely under either of these heads, inasmuch as the irritation is communicated (probably through the medium of the cerebrum,) to some portion of the spinal cord, inducing loss of muscular power, with perhaps excessive sensibility, in the parts which derive their nervous influence from the portion of the cord affected. I would ask whether the effect produced in these cases upon the medulla spinalis is simply irritative, or whether there does not exist an inflammatory action in the *theca* of the implicated nerves at their origin? Dr. Bright has given some very marked and instructive cases of this affection; and under this head my experience would lead me to place the cases of 'spinal irritation' of Dr. Burns, as well as those of 'anomalous affections of the spinal cord,' related by Dr. Abercrombie." 7.

Mr. Prichard relates an example of this kind that occurred in a female 26 years of age, highly susceptible, and who had been long confined to her bed from loss of power in the lower extremities—pain in the abdomen on pressure—dysuria—dysmenorrhœa—leucorrhœa, with a kind of "noli me tangere" sort of susceptibility. The spine was acutely sensible about the last dorsal vertebra. A steady and long-continued treatment directed to the restoration of the uterine function, effected a complete cure.

"I will now advert to Neuralgic Hysteria, properly so called. Cases of extreme local irritability, with perhaps slight, or no other appearance of diseased action, and occurring in females, the disturbance of whose constitution denotes the Hysteric diathesis, are familiar to every practitioner. The part affected may be one of the larger joints, the stomach, or larger bowels, the pleura, or peritonæum, or it may be the mammary gland: in either case, before the irritation has thus fixed itself, it has probably existed in the shape of shifting pains in different parts; the pain, however, once fixed, may remain for a considerable time without any other symptom of diseased action supervening; but, let the state of the general health, and more especially of the uterine system, be disregarded, and we shall find that, sooner or later, the neuralgic may assume the inflammatory stage and its consequences." 8.

Drs. Addison and Bright having given it as their opinion that hysteric irritation never goes on to inflammatory action, our author, with great deference combats this doctrine—and we think not without some success. Exclusive dogmas are seldom found to be just in medicine. Dr. Bright himself admits, that a local congestion may exist in the part which is the seat of hysterical irritation, and the line between irritation and inflammation is often impossible to be distinguished. What, indeed, is the one but a stage of the other? Our author here enters into a rather minute inquiry respecting perforations of the stomach, which he seems inclined to trace to hysterical neuralgia of that organ. He finds, on research, that of the recorded cases of this dreadful accident, the great majority were females. He seems aware, indeed, that the fact of males being subject to perforations of the stomach also, and where hysteria cannot be urged as the cause, is objectionable.

"But such cases, unconnected with scirrhus, or with much constitutional disturbance, are comparatively rare; neither do I consider that their occurrence affects the view which I have here taken of such affections in female subjects; it would be just as unreasonable to argue, that the vomiting of pregnant women is not connected with uterine sympathy, because we have occasion to observe vomiting in the other sex, from other causes. Chronic gastritis will lead to the same result in both sexes, but, I contend that it is of more frequent occurrence in females, and from the causes which I have named." 18.

Mr. P. has drawn up an indiscriminate synopsis of these remarkable cases from such works as were within his reach, and thinks the result bears out his theory. But the list is far from being sufficiently extensive to warrant fully our author's conclusions. We shall conclude with an extract on the treatment of hysteria.

"As regards the Cerebral Hysteria of young females, if we have recourse to an unstimulating diet,—to regular exercise, both of mind and body,—to the use of active aperients, combined with the antispasmodic remedies of former days, —I believe we shall have exhausted our stock of applicable means; a change to that state in which the maternal duties are assumed, may be regarded as certain to remove the evil.

With respect to those anomalous cerebral symptoms which are apt to accompany the cessation of the period, I have only to say, (and I say it with the greatest confidence) that in such cases, the exhibition of tonics (more especially the quinine, and the metallic tonics in general), is calculated to produce the worst effects; to the cerebral excitement already existing, they superadd the stimulus which they unquestionably exercise on the nervous system, and thus greatly aggravate the sufferings of the patient; nor are there wanting cases, in which this excitement, thus produced, has gone on to the production of mania, which has been quickly removed by a recourse to proper treatment. This treatment will be found to consist of active purgation and the occasional application of leeches; it is in these cases that the combination of the fœtid gums with active purgatives, is of especial use.

I proceed to the treatment of Neuralgic Hysteria;—I have attempted to shew that it may present itself in a stage purely Neuralgic or Irritative, or in a more advanced form, which may be defined Organic. In either stage we may look for uterine disorder, (probably of an inflammatory character,) as its cause, if the effect be only irritative, it will be likely to yield to the application of remedies to the exciting cause; if, on the contrary, it is accompanied with inflammatory action, we must also administer that relief which will be found to ensue from local depletion and counter-irritants; in either case, we must carefully investigate the state of the uterus, and if we find evidence of inflammatory action there, our measures must be directed accordingly; the frequent application of leeches to any convenient neighbouring part, (especially at the termination of a scanty and painful period)—local and general warm bathing,—the observance of a recumbent posture,—the rather free exhibition of mercurial alteratives, with mild aperients, an unstimulating diet, and sedative applications locally, will be found among the most useful remedies ; on the other hand, should we detect a want of power in this organ, our remedies must consist of mild tonics, a generous diet, and other means likely to invigorate the system. As a tonic, in such cases, I can speak, from long experience, of the good effect to be derived from the Mistura ferri aromatica of the Dublin Pharmacopœia. It has appeared to me to be nearly free from the stimulating effects to which I have alluded ; I have usually given it in combination with the Dec : Aloe : Comp : and a Pill of Gum : Galbanum and Myrrh, twice a day.

In the treatment of Hysteric gastralgia, it is essential to know whether we have to deal with mere neuralgic irritation, or inflammatory action ; and this observation applies equally to the exciting cause and the effect produced. In this diagnosis, the peculiar habit of the patient, whether plethoric, or atonic ; the character of the countenance, whether flushed or pallid ; the appearance of the tongue, and the state of the uterine function, will be chiefly considered ; or it may be that our suspicion of the inflammatory nature of the disease shall be first awakened by finding the ill effects of tonic remedies, and the contrary relief from local depletion and counter-irritation. Mr. Langston Parker, in his work on the Stomach, places little or no reliance on the appearance of the tongue, as indicative of the state of that organ ; he mentions its pale, flaccid look in some cases, where, after death, inflammation of the mucous membrane was detected. He, however, subsequently observes ' one condition of the tongue may be alluded to, which is almost invariably an index of *gastric inflammation,*—it is when this organ, not materially changed in other circumstances, presents, at the point and edges, a number of vividly red points, resembling grains of vermillion, scattered over the tongue, and appearing to be the papillæ enlarged, and supplied with an encreased quantity of blood ; I believe this condition is seldom found unaccompanied by vascular irritation of stomach.'

There is certainly a state of the tongue, characteristic of uterine disorder, in which it appears pale, flabby, and smooth, or it has, as it were, a soaped appearance down its centre, leaving a margin on either side and the tip, smooth and glazed. But I am inclined to think that where gastric irritation is connected with inflammatory mischief, the tongue will generally be found either as described by Mr. Parker, or with a glazed, moist, pinky surface.

I would here suggest, that, supposing the view which I have taken of Hysteric Gastralgia to be worthy of consideration, it will behove us to adopt a rigid plan of simple diet, and to persevere in local depletion and counter-irritation from the moment that we find the symptoms are not relieved by the usual remedies." 36.

We think Mr. Prichard deserves credit for his inquiry, and hope it may lead to some improvement in the treatment of this troublesome malady.

---

A Fact in the Natural History of Children, hitherto unobserved; which explains much concerning Infantile Diseases and Mortality. By *John Gardner*, Surgeon. 8vo. pp. 23. 1838.

This fact—or fancy—is that in a great many children the brain is unequally developed in all its parts, and that this—" irregular—unequal—accelerated—abnormal, rate of growth of the brain, or of any part of this organ, gives a peculiar character to a child's constitution, rendering it vitally weak, susceptible of morbid impressions, and an easy prey to ordinary diseases." p. 9. This deviation from the healthy development and normal form of the brain, Mr. G. denominates kephalosis, and he avers that, from long experience and observation, he is able to detect these abnormal growths in the heads of children, and gives us several sketches or wood-cuts illustrative of them. Mr. Gardner falls foul of the phrenologists; but we are unable to touch on that delicate subject.

"The age at which kephalosis most prevails is from six months to two or three years; but it may occur at any time from birth to the tenth year, beyond which age my observations upon the changes in the form of the head do not extend. Parents who have sickly children, who have lost a child, or who are anxious to preserve those they possess, will do well to direct their attention to the form and growth of the head at the earliest period, and they will thus be able to discover their tendencies while there is time to avert the danger.

*Signs of Undue Development of Brain, concomitant with the Changes in the Form of the Head.*

10. The integuments and skin of the head in these children have a smooth stretched appearance, and a deep flush follows every slight excitement. The veins are larger and more distinct than usual about the forehead and temples, and easily become full and turgid—as in crying; the head is apt to become hotter than the general surface, and is often bathed in perspiration, especially at night; the hair is frequently redundant and strong, but sometimes it is very deficient.

The fontanelle or open part of the head is large, its edges thin and stretched, and it varies its state of elevation or depression upon slight causes.

The mind of these children is generally premature, their affections lively, and temper quick and exciteable; these qualities render them more than ordinarily interesting. They are more than commonly wakeful, or sleep lightly and are easily disturbed.

Their muscular system is sometimes well developed, as shewn by fine limbs, and an early ability to walk—but this is when the brain does not deviate very seriously from a sound state,—more frequently and in severer cases, the power of walking is protracted, the balancing power of the will being deficient.

A general irritability and exciteability of frame (often deemed characteristic of all children—but much more marked in some than in others, as all persons acquainted with children must have observed) is almost always associated with the condition of head above described. And looking at the offices the brain sustains in the system, there can be no doubt of its depending upon and flowing from it.

At the same time, it will be obvious to every physiologist, how an opposite set of symptoms may flow from a morbid brain, dulness, heaviness, stupor, and a preternatural immobility; but such consequences are comparatively rare." 17.

The *consequences* of these abnormal states are head diseases—especially hydrocephalus, inflammation of brain or membranes, paralysis, croup, &c. The causes of kephalosis, he thinks, are hereditary predisposition, and over-working of the brain itself. The author makes no allusion to the means of counteracting or curing this kephalosis, but as the pamphlet is only the forerunner of a volume on the diseases of children, we shall look to that for more minute details. The brochure is extremely well written, but we cannot help suspecting that the "FACT," after all, is somewhat tinged by fancy.

Practical Compendium of the Materia Medica, with numerous Formulæ, adapted for the Treatment of the Diseases of Infancy and Childhood. By *Alexander Ure*, M.D. Duodecimo, pp. 239. Schloss. 1838.

To Frankel, of Berlin, the author is indebted for the original idea and plan of this little work—as well as for much of the materials of the book. Dr. Ure appears to have directed his attention much to the nature and treatment of infantile diseases, while following the clinical practice and instructions of Barez in Berlin, and Guersent in Paris. In a preliminary dissertation of 70 pages, the author introduces some excellent and judicious directions for the young practitioner, as to bleeding, both general and local—blistering—vomiting—enemata—bathing, &c. drawn from various, and generally the best authorities of this and of foreign countries. Then follows the materia medica, arranged in alphabetical order, according to the last edition of the Pharmacopœia. On this account some of the Continental prescriptions are necessarily modified. It cannot be expected that we should examine, much less analyze the multitude of formulæ in this little manual. Many of them, we confess, are such as will never—or at least, *need* never be used—but the great majority of them are useful prescriptions—and not a few of them will be found new to the English reader. We shall offer the following *specimen* of the work, for reasons which will soon appear.

"ARGENTI NITRAS. *Lunar Caustic* is considered tonic and antispasmodic. It has been given in stomachic epilepsy and chorea, in the dose of 1-8th of a grain, gradually increased to 2 grains. Ryall recommends the external application of a solution of nitrate of silver, (ij. to iij. grains to an ounce of water,) injected betwixt the eyelids, as the most efficient remedy in the blennorrhœal ophthalmia of new-born children. When applied in solution to the mucous membrane of the mouth in the case of aphthæ, or to the fauces in cases of diphtheritis of the pharynx, larynx, or trachea, the new action, induced in the part to which it is applied, according to Dunglisson, extends to the membrane lower down, and exerts at times the most salutary agency. The exudation of coagulable lymph, which has been proved identical with coagulated albumen, and which constitutes the false membrane in cases of diptheritis, frequently begins on the surface of the tonsils, and thence spreads along the arch of the palate, and ultimately descends over the internal surface of the pharynx and œsophagus. According to Dr. Mackenzie, the application of a solution of the nitrate of silver to the tonsils, velum palati, and uvula, frequently removes this plastic exudation, produces manifest relief of the symptoms, and ultimately even dispels them. The solution he uses is 20 grains of the salt to an ounce of distilled water. The astringent effect upon the part of the mucous membrane, with which the solution is made to come in contact, says Dunglisson, may be propagated by continuous sympathy to the part of the trachea lined by the false membrane, a new action may be induced, the albuminoid substance detached, and ultimately thrown off, unless a complete adventitious tube be formed in the trachea, when from the narrowness of the larynx its evacuation is impracticable. Gendron, Stephen Brown, Guimier, found the above mode of cauterization in croup useful, partly as a styptic, partly as a perturbent agent.

Demiron suggested the application of nitrate of silver to small-pox. The pustules are to be punctured, and then touched with a hair-pencil dipped in a solution containing 15 grains to the ounce of water. When the cauterization was performed on the first day, the eruption was seldom developed; the face became swollen, about the seventh day exhibited slight fissures, from which nothing exuded, and the skin desquamated, leaving no perceptible cicatrice, merely a few red spots. When cauterization was performed, on the second day, the formation of pustules, though not arrested, was lessened, and the scars were quite superficial. Beyond the second day, cauterization was inefficient. Velpeau lays open the fully-formed pustules, and cauterizes them along with the adjacent integument with solid nitrate of silver. This method, according to him, checks the development of the eruption, hinders the ophthalmic affection, and moderates the sympathetic disorder of internal organs, especially of the brain. Other practitioners differ on this point. Husson saw death consequent upon cauterization in two cases, and Heller lost a patient from the same cause. Gerardin observed, in the Hospital for children, alarming accidents from the escharotic plan, particularly inflammation of the brain. (*Meissner's Forschungen des 19 Jahrhunderts.* Bd. vi. 1833). Gölis derived advantage from touching the sanguineous tumors of new-born infants, and erectile tumors with the lunar caustic.

A lotion of nitrate of silver of variable strength, according to the degree of local irritation, is a powerful remedy in scaly eruptions of the scalp. The hair ought to be previously removed, and the diseased surface cleansed by means of carrot poultices. An ointment containing 10 grains to the ounce of lard, has been introduced, and advantageously used, by Mr. Guthrie, in chronic inflammation of the conjunctiva. It ought to be recently prepared, and well rubbed in over the diseased surface. The nitrate of silver solution is an admirable application to fungous excrescences and unhealthy sinuous sores. In burns of the first degree, that is, where there is only erythematous redness, the slight contact of the nitrate of silver removes the pain and prevents any subsequent vesication; confluent aphthæ may be treated in a similar manner.

In the dusky red, erysipelatous inflammation existing in the fauces without ulceration or sloughing, the free application of a strong solution of nitrate of silver, (ten grains to the ounce,) to all the parts implicated, is recommended by Messrs. Evanson and Maunsell.

52.

℞ Argenti Nitratis, gr. iss.
Tere cum Micæ panis, q.s.
ut fiat massa in pil.
xij. æquales dividend.
M. *One thrice a day in epilepsy.*

53.

℞ Argent. Nitratis pulveriz., ℈j.

Carbonis fagi, ʒj.

M. ft. Pulvis. *For insufflation upon the eye in blennorrhœal inflammation, with ulcers of the cornea.*

54.

℞ Argenti Nitratis, gr. x.
Ung. Cetacei, ʒj.
Liq. Plumbi diacet., ♏x.
M. *In passive Inflammation of the conjunctiva.*

Guthrie." 22.

Upon the above we have to remark that the nitrate of silver should always be made into pills with extract of liquorice instead of crumb of bread. The dose mentioned in this extract is much too small, especially for adults; but it is a fair dose for children. We shall here mention a curious, and we hope it will turn out to be an important piece of information which has lately come to our knowledge. A gentleman took nitrate of silver in large doses (4 or 5 grains daily) and for much too long a time—several months, for the cure of epilepsy. The cure was effected, but the skin turned intensely blue.* The gentleman, who

---

* We believe that medicines more frequently fail from want of discrimination in the doctor than want of efficacy in the drug. Much discredit has been brought on the nitrate of silver by want of precaution in the prescriber. We lately gave a striking illustration in the case of the blue gentleman of Eaton-square. The present case is another, where a gentleman of talent and merit, but now no more, neglected to warn his patient respecting the duration of the course, and the consequence was that he continued the medicine till the skin turned blue.

was in Scotland at the time, was greatly annoyed by this event, and applied to a country practitioner on the occasion. The medical man (whose name we do not know) advised him to take a drachm of acidum nitricum dilutum, daily in a pint of barley-water, and to sponge his hands and arms twice a day with the same, as a trial, before he applied it to his face. The patient continued this for several weeks, when he applied to the writer of this for advice as to further operations. To our surprise we found the hands and arms nearly as white as they ever had been, while the face and rest of the body were very blue. The patient asserted, however, that his skin was not now nearly so blue as before he used the nitric acid, though it certainly was quite blue enough. The reason of his consulting the writer was to know if the application of the lotion to the face would be attended with any danger. He was assured in the negative, and is now taking the acid and using it externally. He promises to report the result; but there can be little doubt that, as it has removed the blue colour of the hands and arms, it will ultimately restore the natural colour of the face. This will be an important discovery of our northern friend the surgeon.

---

First Principles of Medicine. By *Archibald Billing*, M.D. A.M. &c. Third Edition. London, Highley, 1838.

That a third edition of this book should be now called for, considering that little more than twelve months have elapsed since the second edition appeared, is only what might be anticipated by those acquainted with its merits. The object of the author is to point out the necessity of deducing the treatment of diseases, as far as that can be done, from the ascertained and established laws of life, that is, from physiology. Without such a mode of proceeding the practice of medicine must be neither more nor less than blind empiricism.

"Many persons," says the author (p. 40) "practise well empirically without much brains or reasoning; but he who begins upon principle, and then profits by experience, must be a much more skilful practitioner." The remarks of our author (p. 50.) on the great advantage of combining opium with antiphlogistic measures in the treatment of inflammation, or more correctly speaking, in supporting the system, after the necessary depletion has been employed, when the inflammation is subsiding or past, are excellent; they give the rationale of such treatment very satisfactorily. For the rationale of the treatment of acute and chronic inflammation by the metallic salts (mercury, antimony) and for valuable practical observations on this very important subject, we refer the reader to pp. 64-70.

On the action and nature of stimulants, sedatives, and tonics, and their appropriate employment, see p. 74, &c. The great influence of the nervous system in the production of disease, has been explained most lucidly. In illustrating the nature of morbid, and more especially of inflammatory action, our author has been peculiarly happy. In his illustrations he has taken good care to avail himself of the principal discoveries of modern science, more particularly with respect to the cerebro-spinal and ganglionic systems of nerves. In fact we know of no book which contains, within the same space, so much valuable information, the result not of fanciful theory, nor of idle hypothesis, but of close, persevering clinical observation, accompanied with much soundness of judgment, and extraordinary clinical tact. He has evidently availed himself to the fullest extent of the splendid opportunities he has enjoyed for some years by the situation he holds of physician to the London Hospital, in which capacity, by the way, if we remember rightly, he was the first to introduce the custom of giving regular clinical instruction into the London School of Medicine. We shall terminate our brief notice of the work by earnestly recommending its attentive study to every class of medical students; nor do we think we should be going too far, were we to say that the practitioner will derive much important information and sound practical views of treatment from its perusal. Our advice is,

Indocti discant, ament meminisse periti.

---

OBSERVATIONS ON THE MANAGEMENT OF MADHOUSES, ILLUSTRATED BY OCCURRENCES IN THE WEST-RIDING AND MIDDLESEX ASYLUMS. By *Caleb Crowther*, M.D. Duodecimo, pp. 145. Simpkin and Co.

In this small pocket volume a great number of important questions are discussed; and not a few severe censures levelled at those who will not fail to feel them. Dr. C. sets out by declaiming against the idea and the practice of confining the treatment of insanity or any one disease to an exclusive practitioner on this or that complaint. The following is tolerably caustic.

"We possess excellent monographs of diseases of the heart, lungs, brain, and eyes, but let the reader notice by whom these treatises are written; not by men exclusively attending to one disease, but by men distinguished for their extensive and superior general knowledge. If the superintendent of a madhouse, an oculist, an aurist, or a watering-place doctor should happen to publish on the disease which he pretends to cure, you find that every part of his book, containing information of any value, is pirated from some standard practical work, and that his own composition consists of fabricated cases of cures, of empirical puffing, and of abuse of his rivals."

The following is potassa fusa for some of our fashionables.

"Between confining a medical man's practice to one disease and quackery, there is such a close connection, that I cannot dismiss this subject without making some allusion to it. Of late years, many well-educated medical practitioners, and watering-place doctors, have, in different parts of the kingdom, commenced the general practice of physic upon empirical principles. Divesting themselves of honour, truth, and honesty, their object is to extract from the pockets of their patients the greatest possible quantity of money, in the shortest time possible. For this purpose, they resort to every species of deceit, trickery, and boasting. They congratulate the patient who consults them, upon his fortunate escape from the ignorant treatment of the last person under whose care he has been; and assure him that if he had delayed his application a day or two longer, his life could not have been saved. They endeavour to acquire, secretly, a knowledge of the disease with which the patient about to consult them is affected, and then pretend, intuitively, to have discovered all the patient's ailments from his countenance, and in this manner secure his confidence. In one case, a celebrated spa-doctor told his patient, that on first entering the room, he discovered that the disease respecting which he was about to be consulted was a fetid discharge. A professional man belonging to the church was quite delighted with the doctor's sagacity, and gave him great credit for being able to see a smell."

In the 4th chapter our author inquires whether dysentery in our lunatic asylums be a necessary evil, or the result of negligence? It appears that, out of 13 pauper lunatic asylums, only four reported to the House of Commons that dysentery had spread by infection. Dr. C. is convinced that this disease becomes contagious and diffused by the effluvia of the privies and other excrementitious or filthy matters. We doubt much whether dysentery is ever contagious *per se*, but only when it is an attendant on a low or typhoid *fever*, when the latter may prove infectious, and carry its intestinal character from person to person.

Some severe strictures are made on the election of medical officers to public asylums by magistrates, who are incapable of discriminating talent, and who are, moreover, too often influenced by intrigue and nepotism. Dr. C. has hit Sir William Ellis rather hard, and we think it was neither wise nor charitable in Dr. C. to bring forward the humble origin, and the *res angusta domi* of Sir William in the beginning of his career. There is some truth, but much acerbity in the following passage.

"The physicians met with in ordinary life may be divided into three classes:—the routine, or imperfectly educated; the scientific without, and the scientific with, good practical skill and tact. The routine physicians form a considerable body, and, of course, vary much in their qualifications. Some of them are mere delvers in Mammon's dirty mine. They get into practice not by their medical knowledge, but by the influence of friends and of personal address. Their life is one continued scene of deceit and chicanery, devoted to slandering their superiors and puffing themselves. There are also in this class many good men and useful practitioners, who supply, in a great measure, by close attention, the defect arising from imperfect education and imperfect discriminating powers. Others again, by superior genius and great application, rise from the ranks to the summit of their profession, without passing through the trammels of a regular education. Among the scientific physicians without practical skill or tact, might be enumerated some of the first philosophers of the age. They resemble the mathematician, who understands well not merely the first principles, but the most difficult parts of the science; yet, owing to a want of practical application of his knowledge, and to his ignorance of the use of instruments, he is practically inferior to the midshipman and the civil engineer. Some of our ablest teachers have been formed from this class of physicians. They talk learnedly and elegantly on every subject connected with medicine, but a complicated case of disease, placed under their care, seems to act like a torpedo upon their faculties, and to nonplus them completely. Diffidence, want of nervous energy, of self-possession, and of actual practice in early life, frequently occasion this defect. This class of men, weighing all others by their own standard, are apt to estimate lightly their professional brethren, and to represent them all to be as useless and inefficient as themselves."

This little volume contains numerous remarks which will be found very useful to magistrates, directors, subscribers, and non-medical visitors of lunatic asylums, and to them the book is chiefly addressed. We think it would have been just as well, if the acrimony of the satire had been a little diluted—and especially in the numerous censures which the Doctor has directed against Sir W. Ellis—censures which look like personal pique, though we believe that such does not exist in the Doctor's mind.

---

The Cyclopædia of Anatomy and Physiology. Edited by *Robert Todd*, M.D. F.R.S. &c. &c. Part XIV. Illustrated with numerous Engravings.

Great complaints have been made of delay in the publication of the parts of this Cyclopædia. The present one has been long postponed. The fault, we have every reason to believe, does not lie on the shoulders of the able Editor. Whoseever it may be, it will be for the interest of the work to remedy it.

The contents of this part are:—

Gland *(concluded)*, by R. Dr. Grainger, Esq.—Glosso-pharyngeal Nerve; Dr. John Reid.—Gluteal Region; A.T.S. Dodd, Esq.—Hæmatosine; D. G. O. Rees.—Hand, bones and joints; Dr. Todd.—Hand, Abnormal Condition; R. Adams, Esq.—Hand, Muscles and Regions; F. T. M'Dougall, Esq.—Hearing, Organ of; T. Wharton Jones, Esq.

The execution of all the articles is good. We would particularize that on Gland, by Mr. Grainger—on Hæmatosine, by Dr. Rees—on Abnormal Conditions of the Hand, by Mr. Adams—and on the Organ of Hearing, by Mr. Jones.

It is impossible to give an analysis of these articles. In another place we have alluded to that on Gland, by Mr. Grainger. We shall make an extract or two from the article, and quote the opinions of Mr. Adams.

Speaking of that troublesome accident, dislocation of the first phalanx of the thumb, on the back of the metacarpal bone, Mr. Adams objects to the usually received opinions of the nature of the difficulty in effecting the reduction. He says:—

"The learned author of the First Lines of Surgery has expressed his opinion that the return of the dislocated phalanx to its place is opposed by a combination of causes, viz.—the cuneiform shape of the bone and the resistance of the lateral ligaments, as suggested by Hey, the force of the muscles, and, lastly, he adds, because the surface for the application of the extending means is very limited. To most of these observations we have reason to object, particularly to the last, because we believe that all the force which it is justifiable to use may be easily applied; and we should ever keep in mind a case given on the authority of Mr. Hey, who informs us that the celebrated Mr. Bloomfield reported to his class of pupils at St. George's Hospital, London, that he knew a surgeon increase the force of extension to such a degree in attempting reduction of this dislocation, that he tore off the thumb at the second joint.

The idea that a transverse section of the head of the metacarpal bone presents an outline of a cuneiform figure with the narrowest part of the wedge towards the palm, or forwards, was first advanced by Mr. Hey, and has subsequently been adopted with too little reflection by many writers: for our part we do not think that the head of the metacarpal bone does present this form assigned to it by Hey. But even although it be conceded that it has occasionally a form which would answer to the description given by Mr. Hey, and that its cuneiform figure would facilitate its gliding between the lateral ligaments and forbid its return, surely such an obstacle to the return of the bone would suppose a state of integrity of both lateral ligaments. In our experiments on the dead subject, we found one of these lateral ligaments invariably torn whenever a complete luxation was effected; but with the theory of Hey, which seems to us quite unsupported by the normal anatomy of the bone or the anatomy of the accident, how can we reconcile the observation, that when the first phalanx of the thumb is dislocated to the palmar instead of the dorsal aspect of the metacarpal bone, equal difficulty of reducing the luxation has been experienced by very eminent surgeons! For example, Velpeau says, 'we have seen but once the first phalanx of the thumb pass in front of the first metacarpal bone. The subject of this accident was a woman aged forty-five years; the bone had been out for three days, there was no inflammation.' I thought, says Velpeau, 'that it was owing to some want of skill in myself that I could not succeed in reducing the luxation; but M. Professor Bougon also made fruitless efforts to effect it; finally, M. Roux, with his well-known address and ingenuity, was not more successful, and the bone remained ever afterwards unreduced.'*

Upon the whole it would appear to us that in the case of the dislocation of the first phalanx of the thumb on the dorsum of the metacarpal bone, the cause of the difficulty we experience in reducing it will not be found either in the mechanical resistance of the lateral ligaments or in the interposition of muscular or fibrous parts between the extremities of the dislocated bones, but that, whether the luxation be the common one backwards or the more unusual one forwards, the *vital contraction* of numerous muscles on a small and yielding bone (whose ligaments have been lacerated) will be the principal opposing force we have to contend with."

It is difficult, however, to suppose that mere muscular contraction can oppose so stout a resistance. We doubt the hypothesis of "vital muscular contraction."

* "Velpeau, Traité d'Anatomie des Regions, tom. i. p. 475, edit. 1825.

A Treatise on the Nature and Treatment of Hooping-cough, and its Complications, illustrated by Cases: with an Appendix, containing Hints on the Management of Children, with a view to render them less susceptible of this, and other Diseases of Childhood, in an aggravated Form. By *Geo. Hamilton Roe*, M.D. Octavo, pp. 258. Churchill, August, 1838.

It is now some twenty years or more ago, that our author's attention was drawn to the benefits resulting from prussic acid in hooping-cough. Dr. Roe does not appear to have been then aware of Dr. Granville's work, in which the efficacy of the remedy was adverted to. In his preface, now, he attributes to Dr. G. the merit of having first introduced the remedy into this country for the disease in question. It seems that Dr. Roe was long deterred from recommending prussic acid on account of the prejudices entertained against it; but he now comes forward with confidence to proclaim its powers. There are very few medical practitioners, indeed, who are not in the habit, of late years, of prescribing this medicine daily in many affections of chest and stomach, but in hooping-cough particularly. It would seem that the accidental circumstance of finding prussic acid so useful in pertussis, gradually led Dr. Roe to an especial investigation of the malady itself—and the result is the present volume.

Besides the main part of the work dedicated to hooping-cough, there is a large appendix on the management of children generally. The first part is divided into twelve chapters. We shall glance rapidly at some of these chapters. The first is introductory, and may be passed. The second treats of the nature and progress of hooping-cough. This disease seldom proves fatal per se, but by inducing other affections. The most common is inflammation of the respiratory apparatus. When this is detected by the sagacity of the practitioner, or by auscultation, no time is to be lost, or the patient will sink. A convulsion-fit sometimes comes on even in mild cases, leaving a state of insensibility, interrupted by a succession of fits till one of them terminates life.

Chap. III. details the morbid appearances in hooping-cough. Eleven cases are recorded from various authors—chiefly Watt, Alderson, Laennec—and one or two from the author's own practice, shewing inflammation and its consequences in the different structures of the respiratory apparatus.

### Chap. IV.—Ratio Symptomatum.

As a person very rarely dies of simple hooping-cough, the actual state of the air-passages can only be ascertained by accidental and sudden death from some other disease. Our author has not had such an opportunity. The stethoscope, in the intervals of the paroxysms, merely discloses occasionally a slight râle with the respiratory murmur. The cause of the cough, therefore, must be sought in some functional, not structural affection. Voila the explanation.

"Any one who will make the experiment will perceive, that by the exercise of the voluntary muscles of respiration he cannot either continue coughing loudly for so long a time, or empty the lungs so completely of air, as a person does in a paroxysm of hooping-cough; it must therefore be inferred that the involuntary muscles—namely, those pointed out by Reisseissen as connecting the extremities of the cartilaginous rings of the trachea and bronchiæ—powerfully assist in accomplishing both these objects. They seem by acting spasmodically to expel the air from the lungs, and to excite by sympathy the voluntary muscles of respiration;—the combined action of both sets of muscles appears to produce this peculiar cough."

The cause why the paroxysms are worse after food, is the distention resulting from imperfect digestion. The whoop is occasioned by the violent rush, or rather suction of air through a spasmodically contracted rima glottidis.

The intermission of the paroxysm does not admit of an easy explanation. When the disease goes on unchecked, the chest begins to sound dull, and "either feeble respiratory murmur, or short, and loud respiratory sounds, mixed with large crepitations, are perceived—warning us that the inflammation is communicated to the substance of the lungs, with effusion into the parenchymatous structure, &c." The difficulty of breathing, sense of suffocation, lividity of countenance, blueness of lips, are easily explained in this state of things.

### Chap. V.—Cause and Seat.

It is hardly necessary to say that we know as much of the primary cause of hooping-cough, as of those of measles, cholera, or scarlatina—which is just nothing at all. The spasm, the inflammation, or both, are only the primary—perhaps secondary *effects* of hooping-cough. They have both been advocated as the *causes*. Watt, Alcock, and others have maintained the first hypothesis—Leroy and Webster maintained that the cause of pertussis was in the brain. Inflammation of the pneumo-gastric nerves was accused by Breschet and Herman Kilean; but Dr. Albers, of Bonn, examined the bodies of 47 persons who died of hooping-cough, and in 43 the nervi vagi presented no traces of inflammation—in four, who were scrofulous subjects, the nervus vagus of one side (that on which the body lay) was found reddened. Laennec, Albers, Pinel, and others, look upon spasm as the proximate cause of pertussis. Our author came to the same conclusion as a French writer (Mons. Blache) had done previously, unknown to Dr. Roe.

"The conviction he has expressed as to the nature and seat of the disease (and wherein he coincides substantially with Albers of Brême, Laennec, and Pinel,) is, that hooping-cough must be considered "*as a nervous affection, having its seat both in the mucons membrane of the bronchiæ and in the pneumo-gastric nerves: an affection very frequently complicated with bronchitis and with pneumonia, but which may exist without them; and, like all other diseases of the same kind, having no anatomical marks of any importance.*"

Chap. VI. is a short one, on the contagious character of pertussis. Dr. R. comes to the following conclusion:—

"We may therefore consider that hooping-cough is an epidemic disease, but that it may also be communicated from diseased to healthy individuals by contagion."

### Chap. VII.—Treatment.

Our author very properly remarks, that no specific treatment can be laid down for this disease, seeing the complications with which it may be mixed up. The principal medicines in use hitherto were, opium, lettuce, emetics, acetate of lead, hemlock, arsenic, belladonna, sulphuret of potash, prussic acid, tar vapour—counter-irritation. Upon each of these our author makes remarks—judicious enough—but not necessary to be repeated here. In a chapter on simple pertussis we meet the following passage, which we shall extract.

"I do not wish it to be supposed by my professional brethren that I am recommending hydro-cyanic acid as a cure for every case of hooping-cough, well knowing how ready we all are to believe that what is said to cure everything, in reality cures nothing. Cases of this complaint do occur in which hydro-cyanic acid seems to possess little power over it; for such some remedy is still wanting.

But I may truly say the cases are so very numerous in which this medicine succeeds,—especially if it be given at the commencement of the disease,—and so very few in which it fails of speedily producing a beneficial effect upon the cough, that my first impression on hearing that a child who has been using it is not better is, that the acid cannot have been good. Without recommending any shop in preference to another, I may say, that the acid obtained from certain chemists seldom fails of curing hooping-cough, whilst that obtained from others makes little or no impression on the complaint. Hydro-cyanic acid of Scheele's strength will, if exhibited as soon as the whoop is heard, effect a cure in almost every case of simple hooping-cough. If the disease has been going on for many weeks, its effects are not so immediately felt, but nevertheless it will cure in most instances."

Numerous cases are detailed in this chapter, and also in the next, where pertussis is complicated with bronchitis and other diseases, after which follows a chapter on the general treatment of hooping-cough in regard to air, dress, diet, ablutions, &c. containing judicious instructions.

The Appendix consists of about 50 pages "On the General Management of Children." The maxims laid down are good, though, as our author himself candidly admits—"these rules are by no means new." P. 237. But though true and not new, they are seldom put in force. The nurse, in fact, has more weight in the nursery than the doctor, and his injunctions are as commonly disobeyed as his "stuff" is thrown out of the window, when his back is turned. Although this part of the work is adapted almost exclusively to the study of mothers and nurses, there is much in it which the young medical practitioner would do well to learn. The whole work is creditably executed—the language is good—and the book is entirely free from the least attempt at mystery—much less Charlatannerie.

---

Anatomical Tables; containing concise Descriptions of the Muscles, Ligaments, Fasciæ, Blood-vessels, and Nerves. Intended for the Use of Students. By *Thomas Nunneley*, Lecturer on Anatomy and Physiology in the Leeds School of Medicine; Honorary Secretary to the Leeds Philosophical and Literary Society, &c. &c. Duodecimo, pp. 240. Highley, 1838.

The objects of the author of this useful little work are best stated by himself.

"In the construction of this work one great object has been, that while nothing important on those subjects of which it professes to treat should be omitted; yet, that its size should be such that it may easily be carried about. Occasions frequently arise that a small work would be referred to, when a large work could or would not be consulted; and there are many spare minutes (as between lectures, &c.) which might often be profitably employed by the practice of carrying such books as may readily be consulted, especially such as contain facts, where no lengthened train of reasoning is required, which must necessarily be destroyed by broken and interrupted applications. It is for this reason that the Viscera are not introduced; any description of them, to be useful, must be of considerable length, as their importance depends, not so much upon mere outward forms, as upon their minute texture and relative connexions. The Bones are not described, partly because there already exists more than one manual on them, and it would be impossible to have given a description of them, which should be good for any thing, without considerably extending the size of the book. The manner in which the attachment of muscles to individual bones is given, will, it is presumed, be sufficient to call up a tolerably perfect recollection of them; but, above all, a single glance at the bones themselves, (which every student ought to possess or have access to,) will do more than a lengthened description could. The muscles are presented in more than one tabular form; to understand the muscular system well is considerably more difficult than any other part of anatomy; it, however, repays any application, not only on account of its own importance, but also, because this understood, the course and distribution of the blood-vessels and nerves are most easily comprehended."

We may take the following enumeration of the vessels attached to the clavicle, as a specimen of the author's mode of handling his subject. It is taken at random, and will shew what *kind* of work we have before us.

"The Clavicle has six muscles attached to it; of which four arise, two are inserted.

*Arise the*
*Sterno Cleido Mastoïdeus,*
*Sterno Hyoideus,*
*Pectoralis Major,*
*Deltoides:*
*Inserted the*
*Trapezius,*
*Subclavius.*

The Pectoralis Major is attached to the sternal part of the anterior border of the Clavicle; the Deltoides to the acromial portion of the same border; the Sterno-cleido-mastoideus to the sternal part of the posterior border; the Trapezius to the acromial end of the same border; the Sterno-hyoideus to the sternal extremity; and the Subclavius to the under surface of the clavicle."

Our young friends will find this a very useful little book to carry with them. It will serve as a *refresher.*

---

CHEMISTRY OF ORGANIC BODIES. VEGETABLES. By *Thomas Thomson*, M.D., &c. &c. London, J. B. Baillière, pp. 1,076.

WE hail the appearance of this work with sentiments of unqualified satisfaction. A good account of the chemistry of organic bodies is quite a desideratum in this country—a desideratum which the present and the coming volume seem most likely to supply.

Dr. Thomson informs us, in his preface, that—

"The object of the present volume is, to lay before the British chemical public a pretty full view of the present state of the chemistry of Vegetable Bodies. This branch of the science has made so much progress of late years, that a very wide and inviting field has been laid open. Several hundred new substances have either been discovered, or their characters have been determined with such precision, and their composition investigated with such accuracy, as to give a pretty accurate idea of their constitution, and of their connexion with each other. These ultimate analyses, with a very few exceptions, have been all made upon the continent, and chiefly in Germany and France. British chemists have scarcely entered upon the investigations. Dr. Prout, indeed in 1827, published an analysis of sugar, starch, lignin, and some of the vegetable acids. His results were accurate, but his apparatus was of too complex a nature to warrant trustworthy results in the hands of ordinary experimenters. Within these very few years, Mr. Kane of Dublin, and Dr. Gregory, who had been trained to vegetable analysis in Leibig's laboratory in Giessen, have begun the investigation of vegetable bodies. Already various important facts have been ascertained by them; and, if they continue their investigations, there can be no doubt, from their well known sagacity and dexterity, that many important discoveries in this interesting branch of chemistry will result from them.

The present volume, by exhibiting a pretty complete view of the present state of vegetable chemistry; and, by presenting together the most important facts that have been ascertained, will, I trust, be of some use to those British chemists, who may wish to enter upon this very interesting field of investigation."

Dr. Thomson has deferred until the following volume, on the chemistry of animal substances, a minute detail of the method for obtaining an accurate analysis of vegetable substances. He has been induced to do so, on account of the great size of the present volume; combined with the circumstance that very minute details of that method have been given by Liebig and Dumas, the two chemists to whom we are indebted for the greatest number, and the most accurate vegetable analyses which we possess.

Dr. Thomson has divided his work into four parts, of which the first treats of vegetable principles; the second, of the parts of plants; the third, of vegetation; and the fourth of the decomposition of plants.

The first part comprises 4 classes; 1st. Vegetable Acids, subdivided into volatile acids, fixed and oily acids, acids containing azote, compound acids, and cyanogen and its compounds. 2nd. Vegetable Alkalies, consisting of alkalies analyzed, and alkalies imperfectly examined. 3rd. Intermediate Bodies, divided into alcohol and its compounds, ethers, pyroxylic spirit and its compounds, colouring matters, volatile oils, &c. 4th. Neutral Vegetable Principles, comprising amides, benzoyl and its compounds, spiroil, sugars, gums, caoutchouc, &c. The second Part treats of the sap and gases of plants, barks, roots, bulbs, &c. The third, of the structure, temperature and food of plants, of the motion of the sap, the function of the leaves, and of the decay of plants. The fourth part is divided into venous, panary and acetous fermentation, and putrefaction.

It would, of course, be utterly impossible in any limited space, to give a proper idea of this valuable book, we shall therefore content ourselves with recommending the work to the attention of the public, in the strongest and most energetic manner.

---

### NATURAL HISTORY.

A History of British Birds. By *William Yarrell*, F.L.S., V.P.Z.S. Illustrated by a wood-cut of each species, and numerous vignettes. Parts VII. and VIII.

This pleasant and instructive work goes on spiritedly. When complete it will form an excellent addition to a family as well a professional library. It richly deserves encouragement.

---

A General Outline of the Animal Kingdom. By *Thomas Rymer Jones*, F.Z.S., Professor of Comparative Anatomy in King's College, London. Part I. Price 2s. 6d. Van Voorst, September, 1838.

We are very glad to see this work. It will make an excellent accompaniment to the preceding and to several others of the same cast. Of Mr. Jones's capacity for his task there is no question, and we are sure that this "General Outline" should be in the hands of our medical brethren.

